# KMT2D Induces M1 Macrophage Polarization to Repress Non-small Cell Lung Cancer Progression via Transcription Activation of ITGAL

**DOI:** 10.5812/ijpr-159395

**Published:** 2025-03-08

**Authors:** Wen-Tao Wang, Jie Yang, Peng-Fei Jiang

**Affiliations:** 1 Jingmen Traditional Chinese Medicine Hospital, Hubei Province, China

**Keywords:** NSCLC, Macrophage Polarization, KMT2D, ITGAL

## Abstract

**Background:**

Recent evidence has demonstrated the crucial role of macrophage polarization in promoting non-small cell lung cancer (NSCLC) progression within the tumor microenvironment.

**Objectives:**

This study investigated the possible regulatory mechanism of macrophage polarization during NSCLC development.

**Methods:**

The proportion of M1/M2 macrophages was examined by flow cytometry. The expression of macrophage markers and target molecules was detected by reverse transcription quantitative polymerase chain reaction (RT-qPCR), western blotting, and immunohistochemical staining. Non-small cell lung cancer cells were treated with conditioned medium (CM) from THP-1 macrophages. Cell counting kit-8 (CCK-8), scratch, and transwell assays were used to assess NSCLC cell growth and metastasis. Gene promoter activity was evaluated by dual-luciferase reporter assay. A xenograft model was adopted to determine NSCLC growth in vivo.

**Results:**

Histone-lysine N-methyltransferase 2D (KMT2D) and integrin subunit alpha L (ITGAL) were lowly expressed in NSCLC tissues and cells. The KMT2D overexpression facilitated the polarization of macrophages from M2 to M1 type, which repressed the growth, migration, and invasion of NSCLC cells. Mechanistically, KMT2D promoted the transcription and expression of ITGAL. Inhibition of ITGAL abrogated KMT2D overexpression-mediated M1 macrophage polarization and its anti-cancer effects on NSCLC.

**Conclusions:**

The KMT2D transcriptionally activated ITGAL to trigger M1 macrophage polarization, thereby delaying NSCLC progression. Our findings suggest KMT2D as a potential therapeutic target for NSCLC.

## 1. Background

Non-small-cell lung cancer (NSCLC) is the predominant type of lung cancer, accounting for approximately 80% of lung cancer cases (1). Current therapeutic methods for NSCLC include surgical resection, radiochemotherapy, and targeted therapy (2). Despite significant advancements in diagnosis and therapeutic strategies, most NSCLC patients are diagnosed at an advanced stage, resulting in a poor prognosis (3). Therefore, in-depth research is needed to elucidate underlying mechanisms and identify effective therapeutic targets for NSCLC.

Tumor-associated macrophages represent a major type of immune cell in the tumor microenvironment, playing pivotal roles in the regulation of tumor progression (4). Under different microenvironments, macrophages can be polarized into pro-inflammatory M1 and anti-inflammatory M2 phenotypes (5). Tumor-associated macrophages tend to polarize to an M2-like phenotype, contributing to the malignant development of tumors (6). Therefore, transforming tumor macrophages to the M1 phenotype represents a key strategy for treating NSCLC. However, the regulatory mechanisms for tumor macrophage polarization remain largely unknown.

Histone-lysine N-methyltransferase 2D (KMT2D) is responsible for the methylation of histone H3 at lysine 4 (H3K4me1), playing key roles in the modulation of gene transcription (7). Abnormal expression of KMT2D has been validated in various human cancers, including NSCLC (8). The KMT2D downregulation can accelerate the proliferation and metastasis of NSCLC cells via regulation of the PI3K/AKT/SOX2 axis (8). To date, whether KMT2D is involved in the regulation of tumor macrophage polarization during NSCLC progression has not been reported.

Integrin alpha L (ITGAL) is a member of the integrin family, confirmed to affect the tumor immune microenvironment (9). A recent study found that ITGAL was lowly expressed in NSCLC, which was associated with the immune microenvironment and prognosis of NSCLC patients (10). Notably, the loss of KMT2D was found to decrease ITGAL expression during thymocyte development (11). Thus, we speculated that KMT2D might affect NSCLC development through modulation of ITGAL expression.

Ki-67 is a proliferative gene that facilitates cellular proliferation during all cell cycle phases (G1, S, G2, and mitosis) (12). Ki-67 is widely accepted as an index of cellular proliferation, extensively used to evaluate cell proliferation and growth of various tumors, including lung cancer (13). Therefore, this study detected Ki-67 expression to monitor NSCLC growth in vivo.

## 2. Objectives

Herein, we investigated the role of the KMT2D/ITGAL axis in macrophage polarization during NSCLC progression.

## 3. Methods

### 3.1. Clinical Sample Collection

The NSCLC tissues and paraneoplastic lung tissues were collected from 30 NSCLC patients at the Department of Oncology and Hematology, Jingmen Traditional Chinese Medicine Hospital. The inclusion criteria were as follows: (1) Postoperative pathological diagnosis of NSCLC; (2) age range of 18 - 85 years. The exclusion criteria were as follows: (1) History of other malignant tumors; (2) Receipt of any preoperative treatment; (3) severe impairment of liver or kidney function or severe congestive heart failure; (4) history of cranial or brain injury or trauma. All patients provided informed consent, and the study protocol was approved by the Ethics Committee of Jingmen Traditional Chinese Medicine Hospital.

### 3.2. Reagents and Antibodies

F-12K, RPMI-1640, Airway Epithelial Cell Basal Medium, fetal bovine serum (FBS), lipofectamine 2000, and trizol reagent were purchased from Thermo Fisher (USA). Phorbol myristate acetate (PMA) and RT master mix for qPCR II were purchased from MCE (USA). SYBR Green PCR master mix, BCA Protein Assay Kit, CCK-8 reagent, and Dual-Lucy Assay kit were purchased from Solarbio (Beijing, China). Matrigel was purchased from Sigma (USA) and Corning (USA). Endogenous peroxidase inhibitor, BSA, and DAB were purchased from Phygene (Fuzhou, China). Anti-CD86 (ab239075), anti-Ki67 (ab16667), anti-KMT2D (ab213721), anti-ITGAL (ab307658), anti-β-actin (ab8227), and Goat Anti-Rabbit IgG H&L (HRP) (ab6721) were purchased from Abcam (UK). Anti-CD206 (321101) was purchased from BioLegend (USA).

### 3.3. Cell Culture and Treatment

The NSCLC cell lines (A549, NCI-H1975), normal lung epithelial cells (BEAS-2B), and human monocytic cells (THP-1) were purchased from American Type Culture Collection (USA). Cells were cultured in F-12K medium (for A549 cells), Airway Epithelial Cell Basal Medium (for BEAS-2B cells), or RPMI-1640 medium (for NCI-H1975 and THP-1 cells), supplemented with 10% FBS at 37°C with 5% CO_2_. THP-1 cells were treated with 100 ng/mL PMA for 24 hours to differentiate into macrophage-like cells.

### 3.4. Cell Transfection

Overexpression plasmid for KMT2D, vector, short hairpin RNA targeting ITGAL (shITGAL), and negative control shRNA (shNC) were obtained from GeneChem (Shanghai, China). THP-1 cells were transfected with 4 µg of KMT2D, 4 µg of vector, 2 µg of shITGAL, or 2 µg of shNC using Lipofectamine 2000, according to the manufacturer's protocol. The cells were transfected for 6 hours at 37°C in a cell incubator with 5% CO_2_, followed by replacement with fresh culture medium. The cells were then maintained for 48 hours with 5% CO_2_ at 37°C and collected for subsequent experiments.

### 3.5. Quantitative Real Time Polymerase Chain Reaction

Total RNA was extracted from A549, NCI-H1975, BEAS-2B, and THP-1 cells or tissues using trizol reagent and then reverse transcribed into cDNA using the RT master mix for qPCR II. Subsequently, target gene expression was determined using the SYBR Green PCR master mix on the real-time PCR system (Applied Biosystems, USA). The conditions for PCR cycling were as follows: Activation of TaqMan at 95°C for 10 minutes, followed by 40 cycles of denaturation at 95°C for 10 seconds, and annealing/extension at 60°C for 60 seconds. Gene levels normalized to GAPDH were quantified using the 2^–ΔΔCt^ method. The primers were designed and synthesized by Sangon Biotech (Shanghai, China). The primer sequences are listed in Table 1. 

**Table 1.** Oligonucleotide Primer Sets for RT-qPCR

Name	Sequence (5'-3')	Length
KMT2D		
Forward	GAGCTACGGCGCTTTGAGTT	20
Reverse	AGGGAAACCAATCTGTGATAGGT	23
**ITGAL**		
Forward	TGCTTATCATCATCACGGATGG	22
Reverse	CTCTCCTTGGTCTGAAAATGCT	22
**TNF-α**		
Forward	GTAGCCCATGTTGTAGCAAACC	22
Reverse	TCTGGTAGGAGACGGCGATG	20
**iNOS**		
Forward	CCAATCGACTGCGTTTGTCC	20
Reverse	GATGTCCCAGCCATCGAACA	20
**IL-10**		
Forward	ACACATCAGGGGCTTGCTC	19
Reverse	GAAGTGGGTGCAGCTGTTCT	20
**TGF-β**		
Forward	GGAAATTGAGGGCTTTCGCC	20
Reverse	CCGGTAGTGAACCCGTTGAT	20
**GAPDH**		
Forward	CCAATCGACTGCGTTTGTCC	20
Reverse	GATGTCCCAGCCATCGAACA	20

Abbreviations: RT-qPCR, reverse transcription quantitative polymerase chain reaction; KMT2D, histone-lysine N-methyltransferase 2D; ITGAL, integrin subunit alpha L.

### 3.6. Western Blotting

The tumor tissues and cells were homogenized with RIPA lysis solution (Thermo Fisher) for 30 minutes, followed by centrifugation at 14,000 g for 30 minutes at 4°C. Protein concentration was analyzed using the BCA Protein Assay Kit. Protein samples were mixed with loading buffer and then boiled at 100°C for 10 minutes. The protein samples (30 µg) were loaded onto SDS-PAGE and then transferred to PVDF membranes. After blocking with 5% nonfat milk for 1 hour at 25°C, the membranes were incubated with primary antibodies against KMT2D (ab213721, 1:1000, Abcam, UK), ITGAL (ab307658, 1:1000, Abcam), and β-actin (ab8227, 1:1000, Abcam) overnight at 4°C. Subsequently, the membranes were washed three times and incubated with Goat Anti-Rabbit IgG H&L (HRP) (ab6721, 1:2000, Abcam) for 1 hour at 25°C. Finally, the West Femto ECL Substrate reagent (Solarbio) was used to develop the membranes according to the recommended specifications. Band density was quantified using ImageJ software (National Institutes of Health, USA).

### 3.7. Flow Cytometry

THP-1 cells were collected and subjected to Fc blocking (BioLegend, USA) for 10 minutes. Cells were then stained with anti-CD86 (ab239075, 1:500, Abcam) and anti-CD206 (321101, 1:200, BioLegend) for 30 minutes at 4°C in the dark. Subsequently, the cells were centrifuged at 1,500 g for 5 minutes at 25°C and washed twice. The stained cells were resuspended in PBS and analyzed using a flow cytometer (BD Bioscience, USA).

### 3.8. Cell Counting kit-8 (CCK-8)

The NSCLC cells were inoculated into 96-well plates at a density of 4,000 cells per well. After treatment with conditioned medium (CM) from THP-1 cells for 24, 48, and 72 hours, each well was supplemented with 10 µL of CCK-8 reagent. Following incubation at 37°C for 2 hours, the absorbance was measured at 450 nm using a microplate reader (Thermo Fisher).

### 3.9. Scratch Assay

The migratory capacity of NSCLC cells was assessed using a scratch assay. In brief, 5 × 10^5^ A549 and NCI-H1975 cells were seeded into 6-well plates and maintained until they reached 100% confluency. A 200-µL sterile pipette tip was then used to make a straight scratch in the middle of each well. After washing with PBS to remove cellular debris, the cells were cultured in serum-free medium for 24 hours. Images were captured at 0 hours and 24 hours after the scratch using a microscope (Olympus, Japan). The migration distance was measured using ImageJ software.

### 3.10. Transwell Assay

Transwell chambers with 8 μm pores were precoated with Matrigel at 37°C for 1 hour. The NSCLC cells (2 × 10^4^) were suspended in 200 µL of FBS-free medium and added to the upper chambers, while 500 µL of culture medium containing 10% FBS was added to the lower chambers. After 48 hours of culture, the invaded cells passing through the membrane were fixed with 10% methanol for 20 minutes and then stained with 0.1% crystal violet for 15 minutes at room temperature. The chambers were photographed under a light microscope at five random fields.

### 3.11. Dual Luciferase Reporter Assay

The wild-type (WT) or mutant (Mut) ITGAL promoter fragments containing KMT2D binding sites were amplified and subcloned into the pGL3 plasmid (Promega, USA). Thereafter, THP-1 cells (5 × 10^5^) were seeded in 24-well plates and co-transfected with ITGAL-WT or ITGAL-MUT plasmid, along with either a vector or KMT2D overexpression plasmid, using Lipofectamine 2000 for 24 hours at 37°C. Luciferase activity was tested using the Dual-Lucy assay kit 24 hours after transfection. Renilla luciferase activity was used for normalization.

### 3.12. Xenograft Model

Six-week-old male BALB/c nude mice were purchased from Vital River Laboratory Animal Technology Co., Ltd (Beijing, China), and randomized into the following groups: CM (-) PBS, CM (+) vector, CM (+) KMT2D overexpression, CM (+) KMT2D overexpression + shNC, and CM (+) KMT2D overexpression + shITGAL (n = 6 per group). For xenograft studies, PBS or CM-treated A549 cells (1 × 10^6^ cells per mouse) were injected subcutaneously into the right flank of the mice. The length and width of tumors were recorded, and tumor volume was calculated using the formula: Length × width^2^ × 0.5. The mice were euthanized 30 days after injection. Xenografts were excised, weighed, and photographed. The animal experiments were approved by the Ethics Committee of Jingmen Traditional Chinese Medicine Hospital.

### 3.13. Immunohistochemical Staining

The tumor tissues were fixed with 4% formaldehyde for 24 hours, embedded in paraffin, and cut into 4 μm sections. After dewaxing, the sections were treated with 3% H_2_O_2_ at room temperature for 5 minutes and washed with PBS. The sections were incubated in citrate buffer (pH 6.0) at 95°C for 15 minutes and blocked with 3% BSA for 10 minutes. The sections were then probed with a primary antibody against Ki67 (ab16667, 1:200, Abcam) overnight at 4°C, followed by incubation with a secondary antibody (ab6721, 1:2000, Abcam) for 1 hour at room temperature. After development with DAB for 10 minutes and hematoxylin counterstaining for 3 minutes at room temperature, the sections were observed using a light microscope.

### 3.14. Statistical Analysis

Data are presented as mean ± standard deviation (SD). GraphPad Prism 6.0 was used for statistical analysis, employing Student’s *t*-test for comparisons between two groups or one-way ANOVA for multiple groups. Pearson correlation analysis was used to evaluate the correlation between KMT2D and ITGAL expression. A P-value of less than 0.05 was considered statistically significant.

## 4. Results

### 4.1. Down-Regulation of Histone-Lysine N-Methyltransferase 2D and Integrin Subunit Alpha L in Non-small Cell Lung Cancer Tissues and Cells

**Figure 1.**
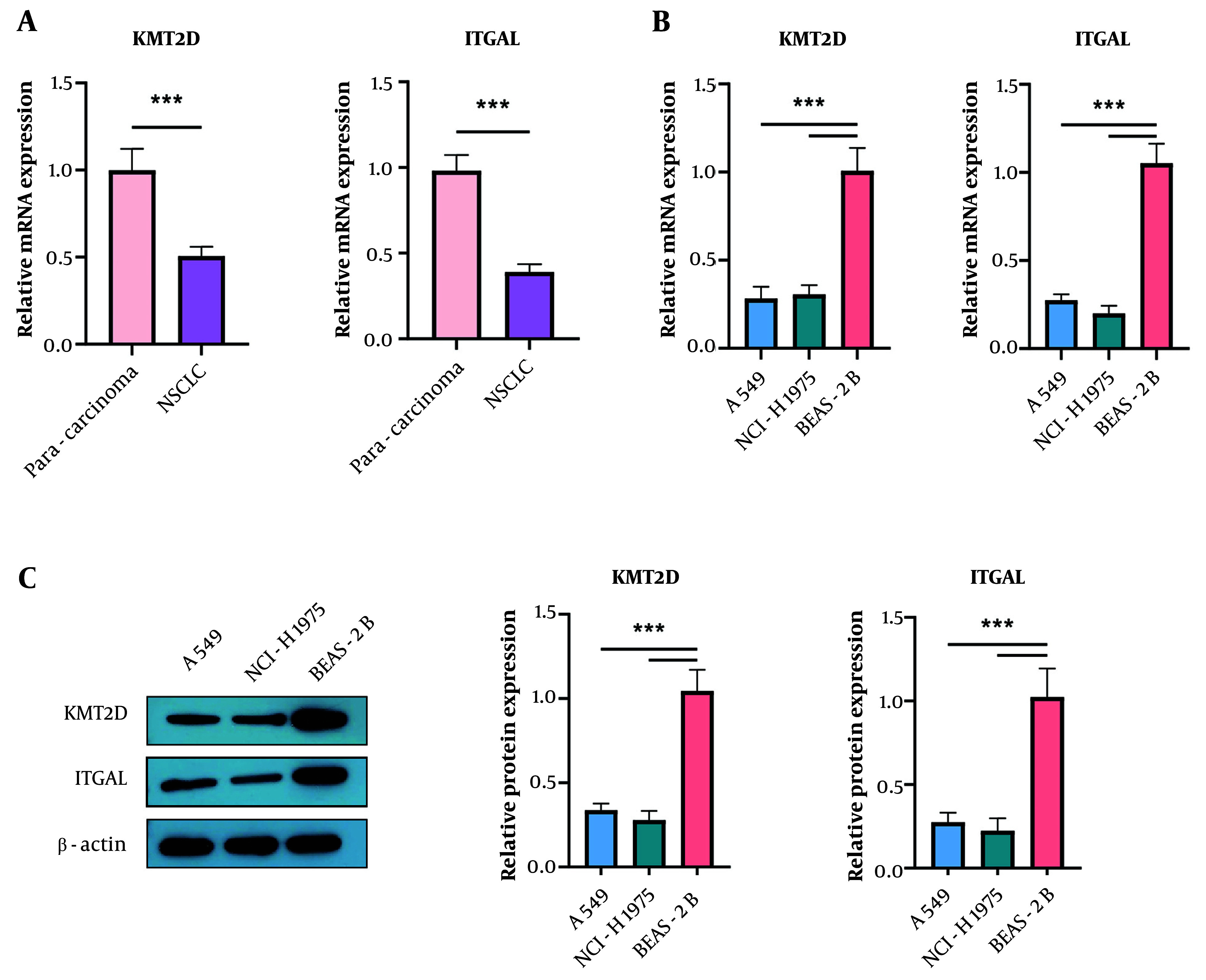
Histone-lysine N-methyltransferase 2D (KMT2D) and integrin subunit alpha L (ITGAL) were lowly expressed in non-small cell lung cancer (NSCLC) tissues and cells. A, reverse transcription quantitative polymerase chain reaction (RT-qPCR) analysis of KMT2D and ITGAL expression in NSCLC tissues and paraneoplastic lung tissues (n = 30); B and C, KMT2D and ITGAL levels in NSCLC cells and normal BEAS-2B cells were assessed by RT-qPCR and Western blotting (n = 3), *** P < 0.001.

First, we examined the differential expression of KMT2D and ITGAL in 30 paired NSCLC and para-cancerous lung tissues. We found that both KMT2D and ITGAL mRNA levels were lower in NSCLC samples compared to para-cancerous normal tissues (KMT2D and ITGAL were lowly expressed in NSCLC tissues and cells. (A) reverse transcription quantitative polymerase chain reaction (RT-qPCR) analysis of KMT2D and ITGAL expression in NSCLC tissues and paraneoplastic lung tissues (n = 30). (B) and (C) KMT2D and ITGAL levels in NSCLC cells and normal BEAS-2B cells were assessed by RT-qPCR and Western blotting (n = 3), (*** P < 0.001), (Figure 1A). Additionally, a reduction in KMT2D and ITGAL mRNA expression was validated in NSCLC cells compared to normal BEAS-2B cells (Figure 1B). Consistently, the protein abundance of KMT2D and ITGAL was decreased in NSCLC cells (Figure 1C). These results demonstrate that KMT2D and ITGAL are abnormally lowly expressed in NSCLC tissues and cells.

### 4.2. Enforced Expression of Histone-Lysine N-Methyltransferase 2D Triggered M1 Phenotype, but Repressed M2 Phenotype Macrophage Polarization

**Figure 2.**
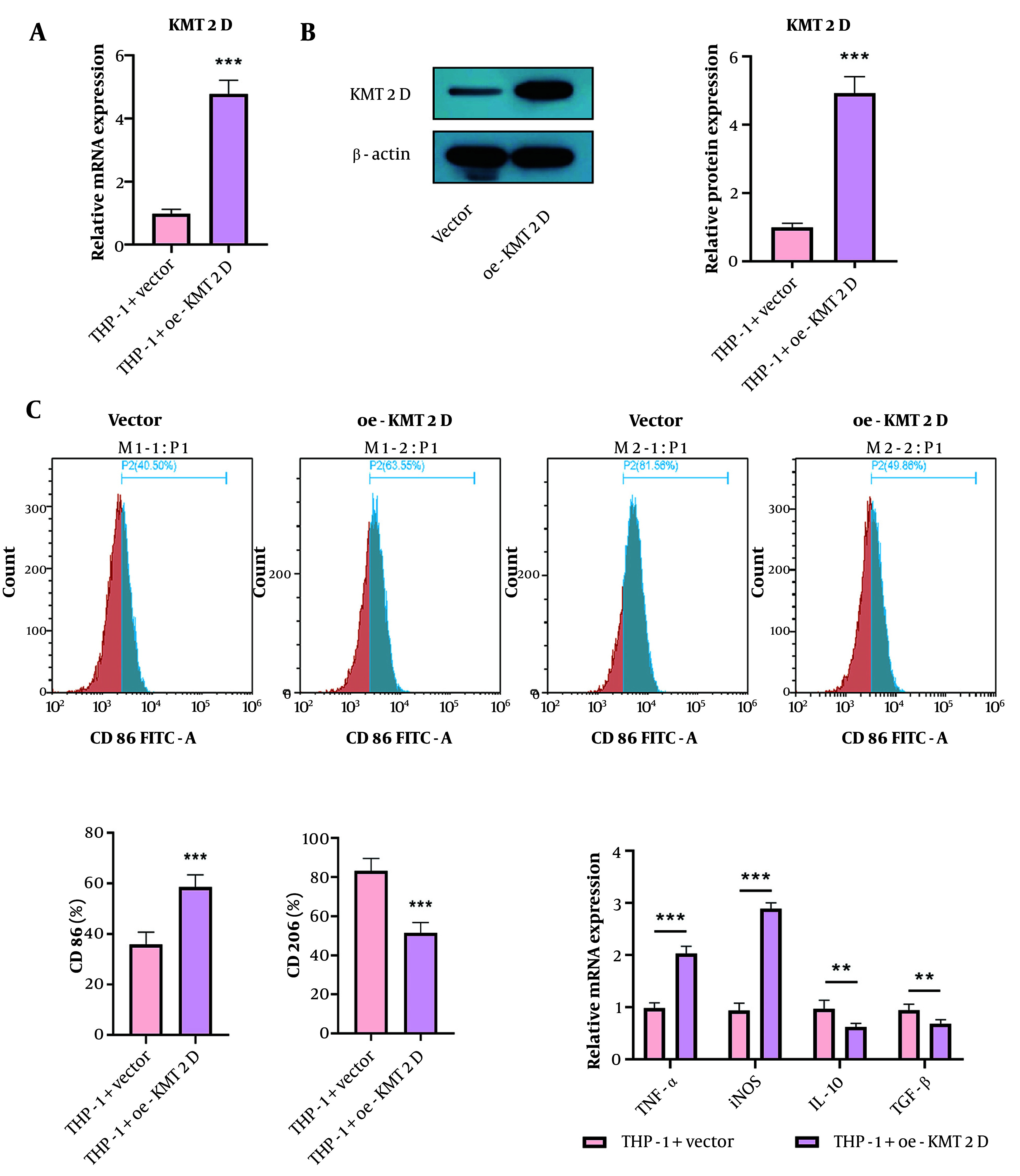
Histone-lysine N-methyltransferase 2D (KMT2D) overexpression triggered M1 phenotype, but repressed M2 phenotype polarization of macrophages. Human monocytic cells (THP-1) were transfected with vector or KMT2D overexpression plasmid. A and B, KMT2D expression in THP-1 cells were detected by reverse transcription quantitative polymerase chain reaction (RT-qPCR) and Western blotting; C, the percentage of M1 macrophages (CD86+) and M2 macrophages (CD206+) were determined by flow cytometry; D, the mRNA levels of M1 markers (TNF-α and iNOS) and M2 markers (IL-10 and TGF-β) were measured by RT-qPCR (n = 3), ** P < 0.01, *** P < 0.001.

To study the influence of KMT2D on macrophage polarization, THP-1 cells were differentiated into M0 macrophages by treatment with PMA, followed by transfection with a KMT2D overexpression plasmid. The overexpression efficiency of KMT2D was validated by RT-qPCR and Western blotting (Figure 2A and B). Functionally, KMT2D overexpression increased the percentage of M1 macrophages (CD86+), while decreasing the percentage of M2 macrophages (CD206+) (Figure 2C). Additionally, the mRNA levels of M1 markers (TNF-α and iNOS) were upregulated, whereas M2 markers (IL-10 and TGF-β) were downregulated in KMT2D-overexpressed THP-1 cells (Figure 2D). These observations suggest that KMT2D overexpression induces macrophage polarization from the M2 to the M1 phenotype.

### 4.3. Histone-Lysine N-Methyltransferase 2D-mediated M1 Macrophage Polarization Restrained Growth and Metastasis of Non-small Cell Lung Cancer Cells

**Figure 3.**
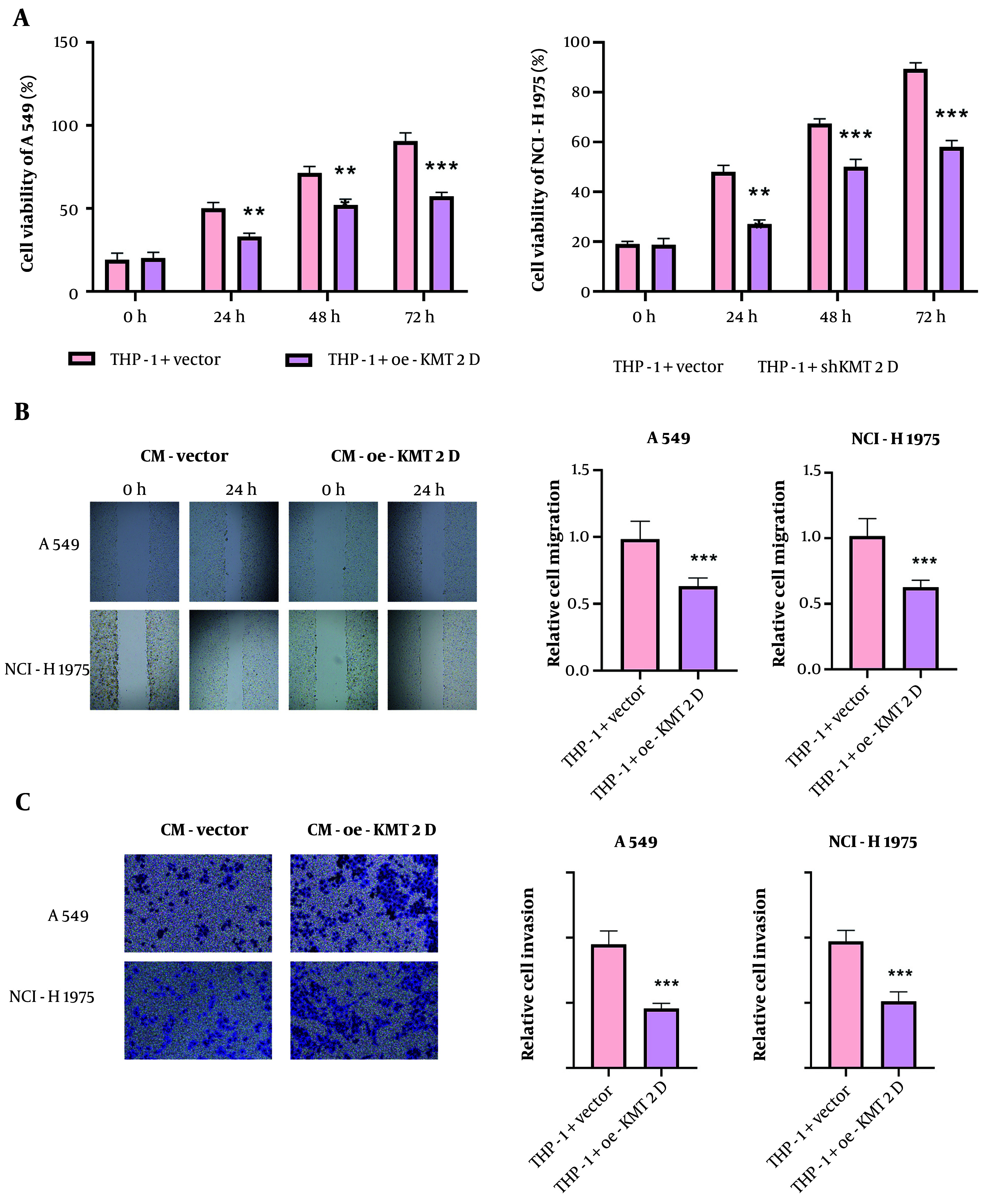
Histone-lysine N-methyltransferase 2D (KMT2D) restrained growth and metastasis of non-small cell lung cancer (NSCLC) cells via inducing M1 macrophage polarization. The NSCLC cells were co-cultured with condition medium (CM) from THP-1 cells transfected with vector or KMT2D overexpression plasmid. A, NSCLC cell proliferation was evaluated by CCK-8; B, migration of NSCLC cells was analyzed by scratch assay; C, the invasive capacity of NSCLC cells was detected by transwell assay (n = 3), ** P < 0.01, *** P < 0.001.

To investigate whether KMT2D affects NSCLC malignant capacities via modulation of macrophage polarization, NSCLC cells were treated with CM collected from KMT2D-overexpressed THP-1 cells. Cell counting kit-8 (CCK-8) data revealed that treatment with CM from the KMT2D-overexpressed group significantly repressed NSCLC cell proliferation (Figure 3A). Moreover, the migration of NSCLC cells was suppressed by administration of CM from THP-1 cells with KMT2D overexpression (Figure 3B). Furthermore, co-culture with CM from KMT2D-overexpressed macrophages markedly inhibited the invasion of NSCLC cells (Figure 3C). In summary, the proliferation, migration, and invasion of NSCLC cells were restrained by KMT2D-mediated M1 macrophage polarization.

### 4.4. Histone-Lysine N-Methyltransferase 2D Induced M1 Macrophage Polarization via Promoting Transcription and Expression of Integrin Subunit Alpha L

**Figure 4.**
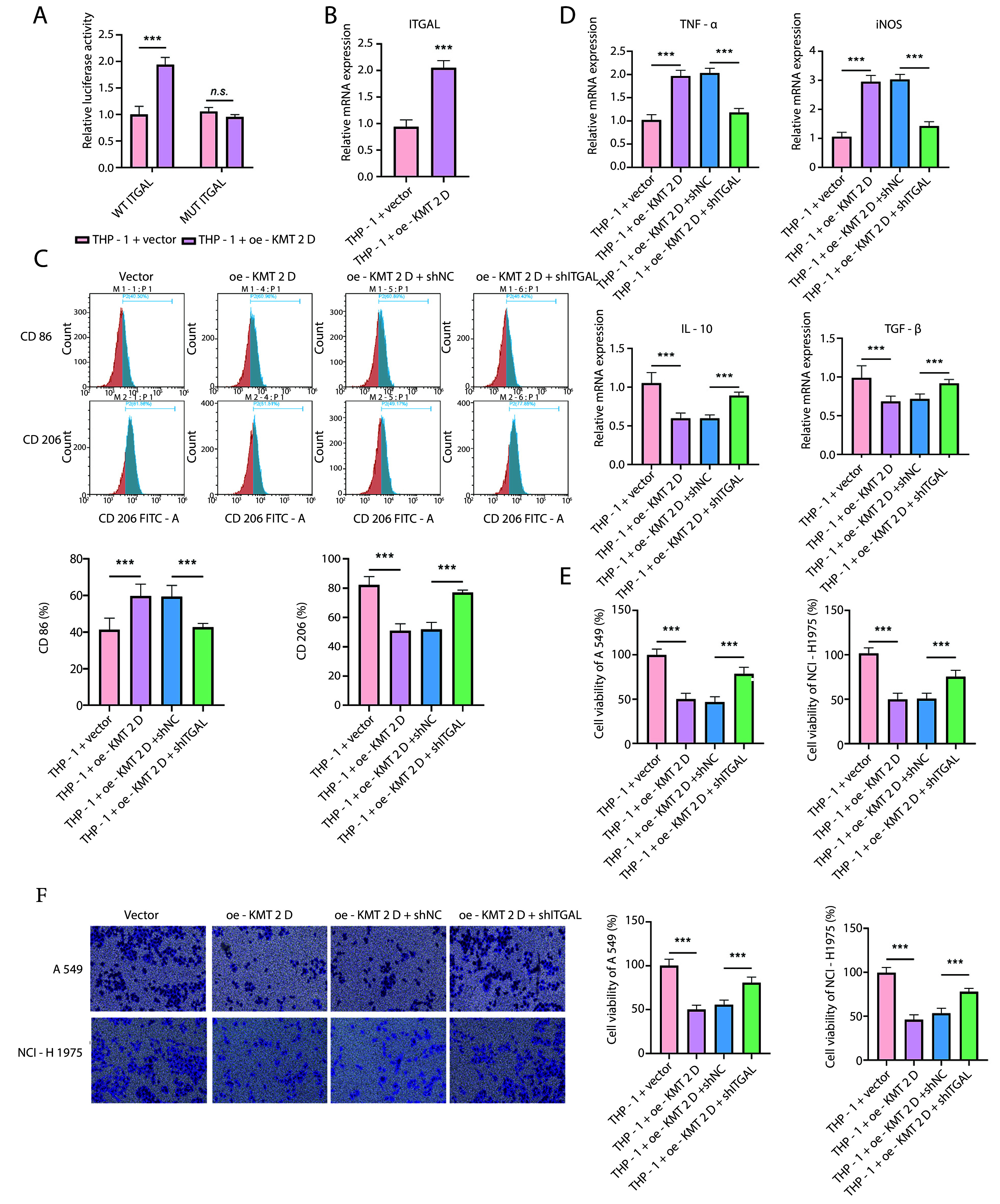
Histone-lysine N-methyltransferase 2D (KMT2D) induced M1 macrophage polarization via promoting transcription and expression of integrin subunit alpha L (ITGAL). A, transcription activity of ITGAL promoter in human monocytic cells (THP-1) transfected with vector or KMT2D overexpression plasmid was analyzed by dual luciferase reporter assay; B, ITGAL mRNA expression in THP-1 cells after transfection with vector or KMT2D overexpression plasmid was assessed by reverse transcription quantitative polymerase chain reaction (RT-qPCR). Human monocytic cells were transfected with KMT2D overexpression plasmid together with or without shITGAL; C, flow cytometry determined the percentage of M1 macrophages (CD86+) and M2 macrophages (CD206+); D, RT-qPCR analysis of the mRNA levels of TNF-α, iNOS, IL-10 and TGF-β. non-small cell lung cancer (NSCLC) cells were co-cultured with condition medium (CM) from THP-1 cells transfected with KMT2D overexpression plasmid in combination with or without shITGAL; E, NSCLC cell proliferation was assessed by CCK-8; F, the invasion of NSCLC cells was analyzed by transwell assay (n = 3) ** P < 0.01, *** P < 0.001.

To further explore the mechanism through which KMT2D regulates macrophage polarization, we focused on ITGAL. The dual-luciferase reporter assay indicated that KMT2D overexpression significantly increased the transcriptional activity of ITGAL (Figure 4A). Accordingly, the ITGAL mRNA level was significantly enhanced in THP-1 cells by KMT2D overexpression (Figure 4B). Flow cytometry showed that the KMT2D overexpression-mediated upregulation of the M1 macrophage proportion and downregulation of the M2 macrophage proportion were reversed by ITGAL silencing (Figure 4C). Additionally, the increased TNF-α and iNOS levels and decreased IL-10 and TGF-β levels in KMT2D-overexpressed THP-1 cells were counteracted by ITGAL knockdown (Figure 4D). Furthermore, the reduced NSCLC cell viability triggered by CM collected from KMT2D-overexpressed THP-1 cells was partly recovered by shITGAL co-transfection (Figure 4E). Moreover, ITGAL depletion abrogated the KMT2D overexpression-mediated inhibition of migration in NSCLC cells treated with CM from THP-1 cells (Figure 4F). Taken together, KMT2D overexpression facilitated the transcription and expression of ITGAL to trigger M1 macrophage polarization, thereby inhibiting the malignant development of NSCLC cells.

### 4.5. Histone-Lysine N-Methyltransferase 2D/Integrin Subunit Alpha L Axis Delayed NSCICL Growth In Vivo Through Inducing M1 Macrophage Polarization

**Figure 5.**
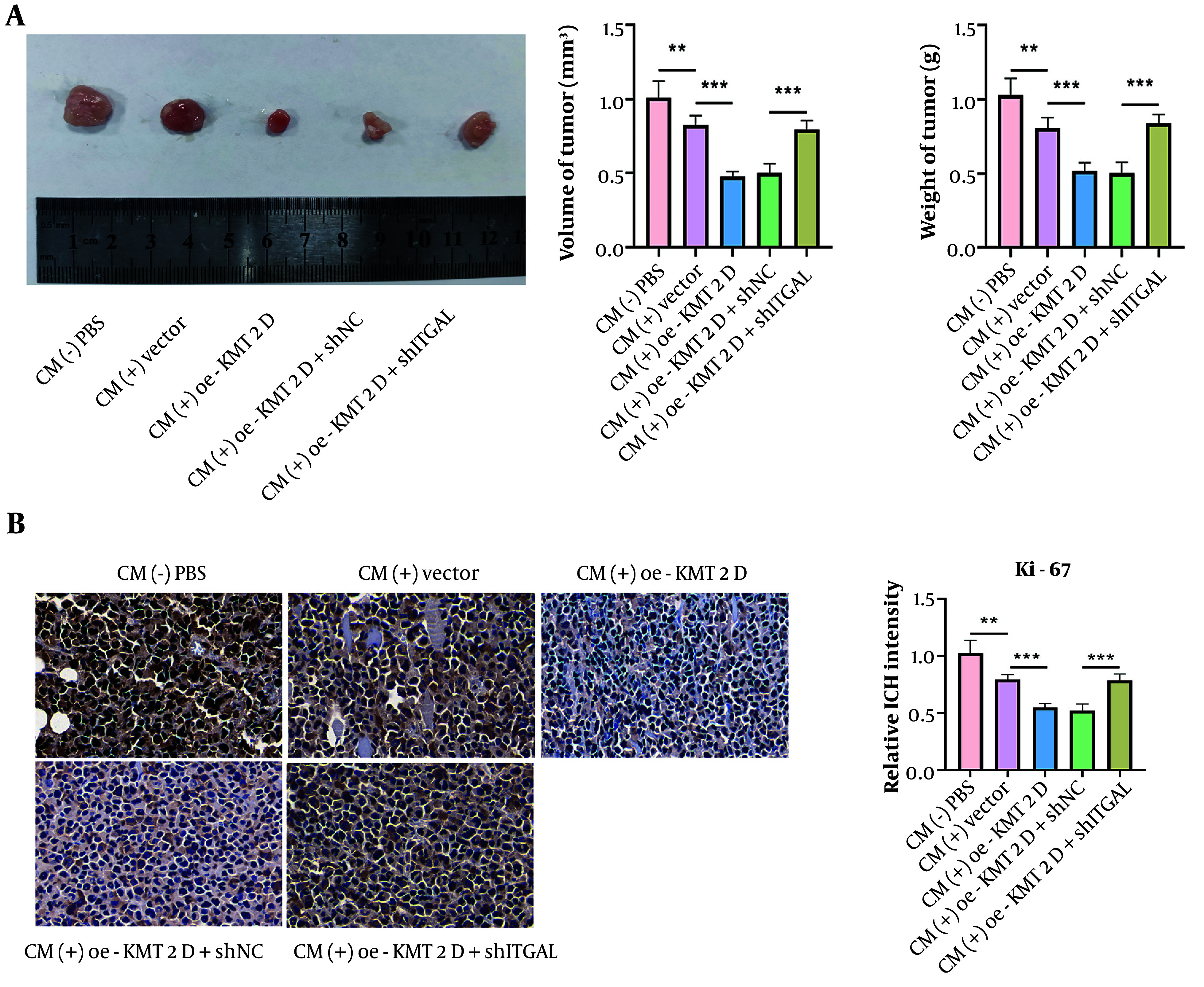
Histone-lysine N-methyltransferase 2D (KMT2D)/integrin subunit alpha L (ITGAL) axis triggered M1 macrophage polarization to inhibit NSCICL growth in vivo. A, representative image of xenografts, tumor volume and weight from different groups were detected; B, expression of Ki-67 in tumor tissues was determined by immunohistochemical staining (n = 6), ** P < 0.01, *** P < 0.001.

Finally, we validated the regulation of the KMT2D/ITGAL axis-mediated M1 macrophage polarization in the in vivo growth of NSCLC cells. As illustrated in Figure 5A, tumor volume and weight were slightly reduced by CM treatment, which was enhanced by KMT2D overexpression, and the anti-cancer effect was abolished by ITGAL deficiency. Additionally, Ki-67 expression was reduced in CM-treated tumors, and this reduction was more pronounced when KMT2D was overexpressed (Figure 5B). However, ITGAL silencing neutralized the KMT2D overexpression-mediated downregulation of Ki-67 expression (Figure 5B). Therefore, the in vivo growth of NSCLC cells was delayed by KMT2D/ITGAL axis-mediated M1 macrophage polarization.

## 5. Discussion

Mounting evidence has demonstrated that inhibition of M2 macrophage polarization can restrain the malignant capacities of NSCLC cells, which is considered an effective intervention for NSCLC (14). In this work, we discovered that KMT2D overexpression transcriptionally activated ITGAL to repress the growth, migration, and invasion of NSCLC cells by shifting macrophage polarization from the M2 to the M1 type. Thus, the KMT2D/ITGAL axis might serve as an effective therapeutic target for NSCLC. As a member of the KMT2 family, KMT2D expression is conserved in eukaryocytes and can promote the transcription of genes through the coordination of methylation of histone H3 lysine 4 (15). Additionally, KMT2D has been suggested to be a key regulator of immune checkpoint blockade in tumors (16). A recent study reported that KMT2D loss contributed to lung cancer growth by enhancing glycolysis (17). Nevertheless, the influence of KMT2D on macrophage polarization during NSCLC progression is poorly understood. Herein, we provided the first evidence that KMT2D triggered M1 type and repressed M2 type polarization of macrophages, which suppressed malignant growth and metastasis of NSCLC cells.

Integrin subunit alpha L has been documented to have a close association with the inflammatory response, which was downregulated by blocking LFA-1 (18). Moreover, ITGAL could also facilitate natural killer cell-induced cytotoxicity (19). Notably, a recent study discovered that ITGAL expression was positively correlated with M1 macrophage infiltration and negatively correlated with M2 macrophage infiltration across various cancers (20), indicating its pivotal role in the tumor immune microenvironment. Wang et al. reported that high expression of ITGAL suggested a better prognosis and delayed proliferation of NSCLC cells (21). In this study, we found lower expression of ITGAL in NSCLC samples and cells. Additionally, ITGAL was found to be downregulated in KMT2D-depleted mice (11), indicating the potential regulation of ITGAL by KMT2D. Our data demonstrated that KMT2D contributed to the transcription and expression of ITGAL in THP-1 macrophages. The ITGAL knockdown counteracted KMT2D-mediated M1 macrophage polarization and its anti-cancer effects on NSCLC.

### 5.1. Conclusions

Taken together, our results indicate that KMT2D promotes ITGAL transcription and expression, which contributes to the shift from M2 to M1 type macrophages, thereby inhibiting NSCLC cell growth and metastasis.

## Data Availability

The dataset presented in the study is available on request from the corresponding author during submission or after publication.

## References

[1] Wu X, Wu J, Dai T, Wang Q, Cai S, Wei X (2024). beta-elemene promotes miR-127-3p maturation, induces NSCLCs autophagy, and enhances macrophage M1 polarization through exosomal communication.. J Pharm Anal..

[2] Laface C, Maselli FM, Santoro AN, Iaia ML, Ambrogio F, Laterza M (2023). The Resistance to EGFR-TKIs in Non-Small Cell Lung Cancer: From Molecular Mechanisms to Clinical Application of New Therapeutic Strategies.. Pharmaceutics..

[3] Yu Y, He J (2013). Molecular classification of non-small-cell lung cancer: diagnosis, individualized treatment, and prognosis.. Front Med..

[4] Shu Y, Cheng P (2020). Targeting tumor-associated macrophages for cancer immunotherapy.. Biochim Biophys Acta Rev Cancer..

[5] Khan F, Pang L, Dunterman M, Lesniak MS, Heimberger AB, Chen P (2023). Macrophages and microglia in glioblastoma: heterogeneity, plasticity, and therapy.. J Clin Invest..

[6] Lin Y, Xu J, Lan H (2019). Tumor-associated macrophages in tumor metastasis: biological roles and clinical therapeutic applications.. J Hematol Oncol..

[7] Yao B, Xing M, Zeng X, Zhang M, Zheng Q, Wang Z (2024). KMT2D-mediated H3K4me1 recruits YBX1 to facilitate triple-negative breast cancer progression through epigenetic activation of c-Myc.. Clin Transl Med..

[8] Yang Y, Qiu R, Weng Q, Xu Z, Song J, Zhao S (2023). MLL4 Regulates the Progression of Non-Small-Cell Lung Cancer by Regulating the PI3K/AKT/SOX2 Axis.. Cancer Res Treat..

[9] Winograd-Katz SE, Fassler R, Geiger B, Legate KR (2014). The integrin adhesome: from genes and proteins to human disease.. Nat Rev Mol Cell Biol..

[10] Zhang R, Zhu G, Li Z, Meng Z, Huang H, Ding C (2024). ITGAL expression in non-small-cell lung cancer tissue and its association with immune infiltrates.. Front Immunol..

[11] Potter SJ, Zhang L, Kotliar M, Wu Y, Schafer C, Stefan K (2024). KMT2D regulates activation, localization, and integrin expression by T-cells.. Front Immunol..

[12] Gerdes J, Li L, Schlueter C, Duchrow M, Wohlenberg C, Gerlach C (1991). Immunobiochemical and molecular biologic characterization of the cell proliferation-associated nuclear antigen that is defined by monoclonal antibody Ki-67.. Am J Pathol..

[13] Shahidi R, Hassannejad E, Baradaran M, Klontzas ME, ShahirEftekhar M, Shojaeshafiei F (2024). Diagnostic performance of radiomics in prediction of Ki-67 index status in non-small cell lung cancer: A systematic review and meta-analysis.. J Med Imaging Radiat Sci..

[14] Sedighzadeh SS, Khoshbin AP, Razi S, Keshavarz-Fathi M, Rezaei N (2021). A narrative review of tumor-associated macrophages in lung cancer: regulation of macrophage polarization and therapeutic implications.. Transl Lung Cancer Res..

[15] Rao RC, Dou Y (2015). Hijacked in cancer: the KMT2 (MLL) family of methyltransferases.. Nat Rev Cancer..

[16] Wang G, Chow RD, Zhu L, Bai Z, Ye L, Zhang F (2020). CRISPR-GEMM Pooled Mutagenic Screening Identifies KMT2D as a Major Modulator of Immune Checkpoint Blockade.. Cancer Discov..

[17] Alam H, Tang M, Maitituoheti M, Dhar SS, Kumar M, Han CY (2020). KMT2D Deficiency Impairs Super-Enhancers to Confer a Glycolytic Vulnerability in Lung Cancer.. Cancer Cell..

[18] Whitcup SM, Chan CC, Kozhich AT, Magone MT (1999). Blocking ICAM-1 (CD54) and LFA-1 (CD11a) inhibits experimental allergic conjunctivitis.. Clin Immunol..

[19] Barber DF, Faure M, Long EO (2004). LFA-1 contributes an early signal for NK cell cytotoxicity.. J Immunol..

[20] Lin F, Yang H, Huang Z, Li Y, Ding Q, Ye Y (2024). Magnesium-related gene ITGAL: a key immunotherapy predictor and prognostic biomarker in pan-cancer.. Front Pharmacol..

[21] Wang Q, Xiao G, Li N, Jiang X, Li C (2023). lncRNA PCBP1-AS1 mediated downregulation of ITGAL as a prognostic biomarker in lung adenocarcinoma.. Aging (Albany NY)..

